# Antimicrobial Activity of Lactic Acid Bacteria Starters against Acid Tolerant, Antibiotic Resistant, and Potentially Virulent *E. coli* Isolated from a Fermented Sorghum-Millet Beverage

**DOI:** 10.1155/2019/2013539

**Published:** 2019-12-14

**Authors:** Stellah Byakika, Ivan Muzira Mukisa, Robert Mugabi, Charles Muyanja

**Affiliations:** Department of Food Technology and Nutrition, School of Food Technology Nutrition and Bioengineering, College of Agricultural and Environmental Sciences, Makerere University, P.O. Box 7062, Kampala, Uganda

## Abstract

Bacterial contamination of fermented foods is a serious global food safety challenge that requires effective control strategies. This study characterized presumptive *E. coli* isolated from *Obushera*, a traditional fermented cereal beverage from Uganda. Thereafter, the antimicrobial effect of lactic acid bacteria (LAB) previously isolated from *Obushera,* against the *E. coli*, was examined. The presumptive *E. coli* was incubated in brain heart infusion broth (pH = 3.6) at 25°C for 48 h. The most acid-stable strains were clustered using (GTG)_5_ rep-PCR fingerprinting and identified using 16S rRNA sequencing. *E. coli* was screened for *Shiga* toxins (*Stx 1* and *Stx 2*) and *Intimin* (*eae*) virulence genes as well as antibiotic resistance. The spot-on-the-lawn method was used to evaluate antimicrobial activity. Eighteen isolates were acid stable and are identified as *E. coli*, *Shigella*, and *Lysinibacillus.* The *Stx* 2 gene and antibiotic resistance were detected in some *E*. *coli* isolates. The LAB were antagonistic against the *E. coli*. Lactic acid bacteria from traditional fermented foods can be applied in food processing to inhibit pathogens. *Obushera* lactic acid bacteria could be used to improve the safety of fermented foods.

## 1. Introduction

There is an increased consumption of fermented cereal-based foods such as *Obushera* [1]. *Obushera* is a traditionally fermented sorghum and/or millet beverage from Uganda. Traditionally, the beverage is spontaneously fermented predominantly by lactic acid bacteria (LAB) [2]. However, given the challenges of spontaneous fermentations, pure starters like *Lactobacillus* (*Lb.*) *plantarum* MNC 21, *Lactococcus* (*L.*) *lactis* MNC 24, and *Weisella* (*W.*) *confusa* MNC 20 were developed [2, 3]. These starters are rapid and excellent lactic acid producers that can lower the pH to <4.5 in just 12 h of fermentation. The resultant lactic acid produced in such fermentations can inhibit pathogens [4, 5]. In fact, rapid product acidification (pH ≤ 4.0) in lactic-fermented products is recommended since it is very inhibitory to pathogens [6]. However, it appears that some foodborne pathogens are very acid tolerant. Recently, Byakika et al. [1] reported the presence of presumptive *E. coli* in *Obushera* (pH ≤ 4.0 and titratable acidity = 0.1–3.1%). Indeed, several reports of undesirable microorganisms in various acidic foods exist [7–11]. This is of great concern because the tolerance to acid stress by pathogens aggravates their virulence [12, 13].

Outbreaks involving acidic foods have increased the attention given to the acid tolerance properties of pathogens [14]. Bacteria may acquire acid tolerance by horizontal gene transfer [15]. Upon exposure to low pH, they use an acid-induced tolerance response (ATR) to survive [16, 17]. The ATR is a phenomenon where microorganisms show increased resistance to acid stress following the exposure to mildly acidic environments [18, 19]. So, for adequate pathogen inhibition, high LAB counts are needed to rapidly lower the pH below 4 [20–22]. Given the increasing demand for safe foods, cultures that are inhibitory to foodborne pathogens are inevitable, more so, since the successful use of antibiotics in the treatment of foodborne illnesses is no longer guaranteed. This is due to overuse and misuse of antibiotics which have created resistance among pathogens [23]. Therefore, this study evaluated the antimicrobial effect of *Lb. plantarum* MNC 21, *L. lactis* MNC 24, *W. confusa* MNC 20, and *Lb. rhamnosus* yoba 2012 against acid-resistant, antibiotic-resistant and potentially pathogenic *E*. *coli* isolated from *Obushera*.

## 2. Materials and Methods

### 2.1. Materials

#### 2.1.1. Lactic Acid Bacteria

*Lb. plantarum* MNC 21 (Gene bank accession number: JF512470), *L. lactis* MNC 24 (Gene bank accession number: JF512471), and *W. confusa* MNC 20 (Gene bank accession number: JQ754455) were isolated from *Obushera* [2]. *Lb. rhamnosus* yoba 2012 (originally named *Lb. rhamnosus* GG) (Yoba for Life Foundation Amsterdam, the Netherlands) was obtained from the Uganda Industrial Research Institute (IURI), Kampala, Uganda. Stock cultures were stored at −20°C in Ringer's solution containing 15% glycerol. The LAB strains were independently propagated according to the procedure described by Mukisa et al. [3]. Briefly, from the stock cultures, 0.1 mL of each strain was separately delivered into 100 mL of sterile MRS broth (Laboratorios CONDA, Madrid, Spain) and incubated anaerobically at 30°C for 24 h. The cells were washed and recovered by centrifugation (7,500 ×g for 10 min). The cell pellets were suspended in 100 mL of sterile Ringer's solution (Oxoid Ltd, Basingstoke, Hampshire, England).

#### 2.1.2. *E. coli*

Presumptive *E. coli* (*n* = 32) previously isolated from *Obushera* by Byakika et al. [1] was used. From the stock cultures, 0.1 mL was separately inoculated in 100 mL of sterile brain heart infusion (BHI) broth (Laboratorios CONDA, Madrid, Spain) and incubated at 30°C for 24 h. The cells were washed and recovered by centrifugation (7,500 ×g for 10 min). The cell pellets were suspended in 10 mL of sterile Ringer's solution (Oxoid Ltd., Basingstoke, Hampshire, England).

### 2.2. Biochemical Characterization

The isolates were characterized by Gram staining, catalase, oxidase, and indole tests using standard methods.

### 2.3. Acid Tolerance

The acid tolerance of the presumptive *E. coli* isolates was determined by adding each cell suspension to 10 mL of lactate acidified BHI broth (pH = 3.6, titratable acidity = 1.5%) to give a final cell concentration of about 10^7^ cfu/mL. The broth was incubated at 25°C. The cells were enumerated at intervals of 0, 24, and 48 h. *E. coli* was enumerated by pour plating using *E. coli*-coliforms chromogenic agar (Laboratorios CONDA, Madrid, Spain) and incubating at 37°C for 24 h. Only isolates that survived the acidified broth for up to 48 h were used for further analyses.

### 2.4. DNA Extraction, PCR, and Sequencing

Genomic DNA of the pure presumptive acid-tolerant *E. coli* isolates was extracted from pure colonies using the GenElute bacterial genomic DNA kit (Sigma-Aldrich, St. Louis, Missouri, USA) following manufacturer's instructions. The extracted DNA was used for (1) (GTG)_5_-Rep-PCR, (2) detection of virulence genes, and (3) 16S rRNA sequencing.

For the (GTG)_5_-Rep-PCR, the protocol was carried out following the manufacturer's instructions. The 25 *μ*L reaction consisted of 12.5 *μ*L 1x master mix with standard buffer (New England Biolabs Inc., MA, U.S.A), 0.2 *μ*M (GTG)_5_ primer (5′GTGGTGGTGGTGGTG3′) supplied by Macrogen, Inc., Seoul, South Korea, 2 *μ*L DNA template, and sterile nuclease-free water. The amplification conditions were as follows: initial denaturation at 95°C for 10 min, 35 cycles of 95°C for 30 s, 40°C for 1 min, 65°C for 3 min, and a final elongation step at 65°C for 8 min. The amplified DNA products were analyzed by electrophoresis in a 2% agarose gel (Sigma-Aldrich, St. Louis, Missouri, USA). The gel was visualized by a UV transilluminator (Syngene G: Box gel documentation system, Fredrick, MD, USA). To generate the dendrogram, TIFF image analysis was carried out using GelJ version 1.0 software [24]. Similarities were calculated using the DICE correlation coefficient and the unweighted pair group method with arithmetic mean (UPGMA). Based on results from the dendrogram, representative strains were selected from each group that indicated similarity in the banding patterns.

Amplification of the 16S rRNA gene was carried out as described by Mukisa et al. [2]. Universal primers 1F (5′GAGTTTGATCCTGGCTCAG3′) and 5R/1492R (5′ GGTTACCTTGTTACGACTT 3′) supplied by Macrogen, Inc., Seoul, South Korea, were used. The PCR was set up in a total volume of 50 *μ*L comprising of 0.2 *μ*M of each primer, 25 *μ*L 1x master mix with standard buffer (New England Biolabs Inc., MA, U.S.A), 4 *μ*L DNA, and sterile nuclease-free water. The initial denaturation step was performed at 94°C for 3 min, followed by 30 cycles of denaturation (94°C, 30 s), annealing (55°C, 30 s), extension (72°C, 3 min), and final extension (72°C, 10 min). The extracted DNA was purified using a QIAquick PCR purification kit (Qiagen, Hilden, Germany) following the manufacturer's instructions. The pure DNA was sequenced with the same primers, using the BigDye® Terminator v3.1 Cycle sequencing kit (Applied Biosystems) and ABI 3730xl DNA analyzer (Applied Biosystems). Sequencing was performed by Macrogen Europe, Amsterdam, the Netherlands, and the identification was done by performing a nucleotide sequence database search at National Centre for Biotechnology Information (NCBI) using the Basic Local Alignment Search Tool (BLAST) program.

The DNA of the presumptive *E. coli* isolates was screened for presence of virulence genes: Shigatoxin I (*Stx 1*), Shigatoxin 2 (*Stx 2*), and intimin (*eae*). The 50 *μ*L reaction consisted of 25 *μ*L, 1X master mix with standard buffer (New England Biolabs Inc, MA, U.S.A.), 0.2 *μ*M each of forward and reverse virulence gene primers, 4 *μ*L DNA template, and sterile nuclease-free water.  Table 1 shows the primers and PCR conditions used.

### 2.5. Antibiotic Resistance

**Figure 1:**
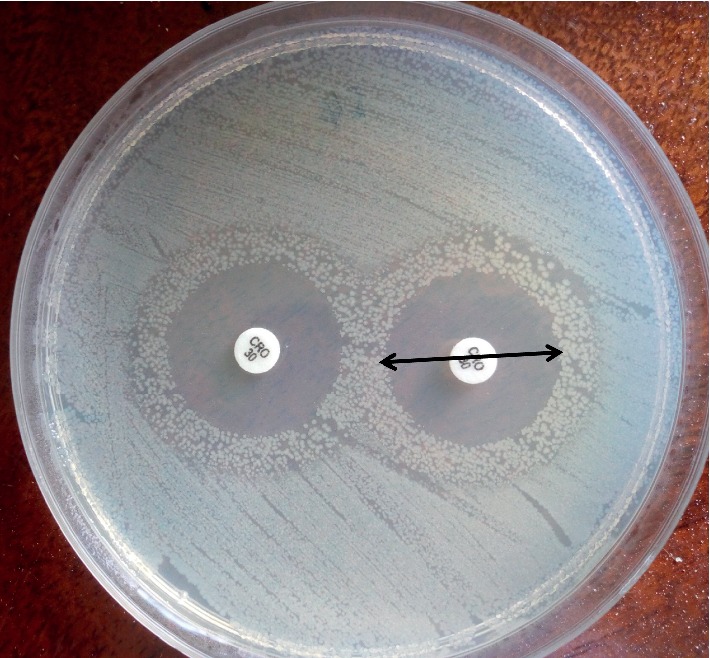
Measurement of the inhibition zone diameter to determine antibiotic susceptibility/resistance using the Kirby–Bauer disk diffusion method. Image showing inhibition zones of ceftriaxone (30 *μ*g) against *E. coli* BMC 4.

The susceptibility of the confirmed *E. coli* isolates to thirteen antibiotics was examined according to the Kirby–Bauer disk diffusion method [27]. The antibiotics were obtained from Bioanalyse (Ankara, Turkey) and included ampicillin (10 *μ*g), amoxicillin (25 *μ*g), amoxicillin-clavulanic acid (30 *μ*g), cephalexin (30 *μ*g), ceftriaxone (30 *μ*g), gentamicin (10 *μ*g), kanamycin (30 *μ*g), tetracycline (30 *μ*g), chloramphenicol (30 *μ*g), ciprofloxacin (5 *μ*g), levofloxacin (15 *μ*g), trimethoprim-sulphamethoxazole (25 *μ*g), and nitrofurantoin (300 *μ*g). For the test, fresh culture suspensions were standardised to 0.5 McFarland (equivalent to 8 log cfu/mL). Using sterile cotton swabs, sterile prepoured plate count agar (PCA) (Laboratorios CONDA, Madrid, Spain) plates were swabbed with standardised culture suspensions and incubated at 37°C for 1 h. Antibiotic discs were then placed on the agar surface and incubated at 37°C for 24 h. The diameter of the inhibition zone was measured in mm (Figure 1). Isolates were categorized as resistant, intermediate, or susceptible according to the guidelines of the Clinical and Laboratory Standard Institute [27]. The multiple antibiotic resistance (MAR) index was computed as *a*/*b*, where *a* is the number of antibiotics the isolate was resistant to and *b* is the total number of antibiotics to which the isolate was exposed [28].

### 2.6. Antimicrobial Activity

**Figure 2:**
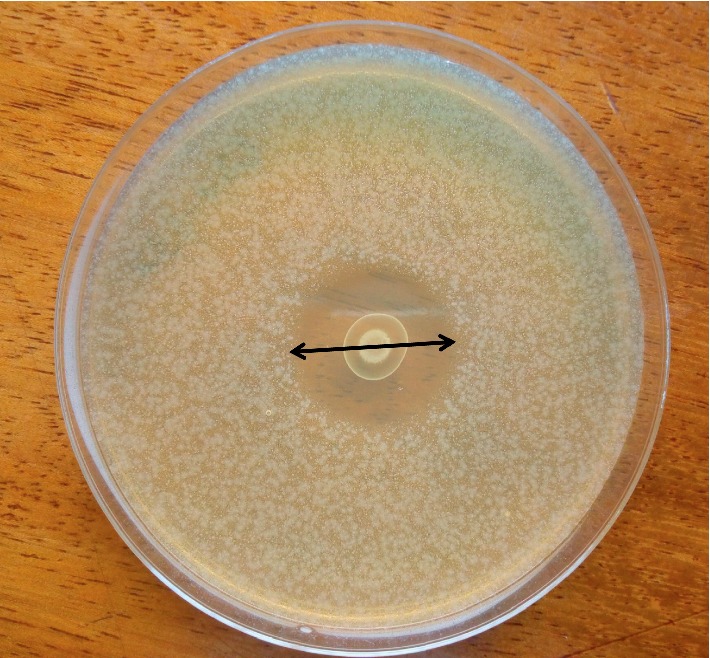
Measurement of inhibition zone diameter to determine antimicrobial activity using the spot-on-the lawn method. Image showing the inhibition zone of *Lb. plantarum* MNC 21 (colony in the center) against *E. coli* BMC 4.

The antimicrobial activity of the LAB starters against *E. coli* was tested using the spot-on-the lawn method as described by Byaruhanga et al. [29] with a few modifications. Briefly, sterile prepoured plate count agar (Laboratorios CONDA, Madrid, Spain) plates were spotted with 10*μ*L of 6 log cfu/mL of each LAB and incubated at 30°C for 24 h. About 10 mL of molten PCA (45°C) seeded with 4 log cfu/mL of *E. coli* was used as the overlay medium and incubated at 30°C for 24 h. The level of inhibition was determined by measuring the diameter (mm) of zone of clearing around the producer colonies (Figure 2).

### 2.7. Statistical Analyses

The data were analyzed using one-way Analysis of variance (ANOVA) to test for significant differences (*p* < 0.05) among treatments. Mean comparisons were done using the least significant difference (LSD). The statistical analyses were done using XLSTAT software (version 2010.5.02, Addinsoft, France).

## 3. Results and Discussion

### 3.1. Biochemical Characterization of Presumptive *E. coli* Isolates

Results showed that all the presumptive isolates were Gram negative, indole positive, catalase positive, and oxidase negative which were typical of *E. coli* [30].

### 3.2. Acid-Tolerant Strains

**Figure 3:**
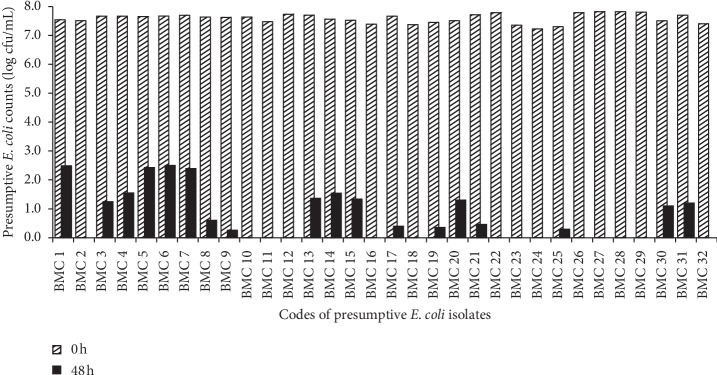
Changes in counts of presumptive *E. coli* between 0 and 48 h of incubation in acidified brain heart infusion broth.

Figure 3 shows the counts of presumptive *E. coli* counts in BHI broth (pH = 3.6, T.A = 1.5%) at 0 and 48 h. The average initial cell count was 7.6 log cfu/mL. At 48 h, counts ranged between 0 and 2.5 log cfu/mL with only 18 out of 32 still detectable.

At pH = 3.6, the main inhibitory component in the BHI broth was lactic acid (titratable acidity = 1.5%). Acid levels in some foods could exceed 1% (w/v) in some foods resulting in an ultimate pH of 3.5–4.5 [31, 32]. According to Raybaudi-Massilia et al. [4] and Davidson [33], organic acids inhibit pathogens by entering into cells in an undissociated form and dissociating within the cytoplasm. This lowers the intracellular pH, and to maintain balance, the cell uses ATP to expel the excess hydrogen ions. This exhausts the cell of energy required for growth and other metabolic processes resulting in death. To counteract this, some pathogens employ ATR to survive acid stress. This acid adaptation involves initial sublethal acid shock resulting in changes in gene expression. There is upregulation of numerous rescue proteins: *F*_0_*F*_1_-ATPase, glutamic acid decarboxylase, groEL, groES, and *σ* factors all of which shield the pathogens from the lethal effects of the acid [34–36]. Several authors have implicated ATR in the facilitation of pathogen virulence [36–38]. Leyer et al. [8] noted the acid tolerance of *E. coli* O157 : H7 as a key factor in its virulence because it protected the cells from the lethal effects of gastric acid. In another study, Gorden and Small [39] observed more acid tolerance among enteroinvasive and enteropathogenic *E. coli* than in the nonpathogenic strains. Acid adaptation is not only problematic in facilitating virulence but also induces cross-protection against other environmental stresses such as heat and salt that may be encountered in food processing [40, 41]. Shen et al. [42] noted that acid adaptation reduced the susceptibility of *Salmonella Typhimurium* to low temperature and other detrimental factors in lactic-fermented milk products.

In this study, the presumptive *E. coli* was isolated from *Obushera* (pH = 3.2–5.6; T.A = 0.1–3.1%) Byakika et al. [1] showed acid tolerance but to varying extents (Figure 3). It is postulated that some of the isolates could have employed the ATR to survive in the acidified beverage. The ATR could have been triggered during fermentation of the beverage because substrate acidification is a gradual process that could facilitate adaptation. In contrast, inoculation in BHI broth (pH = 3.6, T.A = 1.5%) without prior exposure to mild acid conditions could explain the rapid inhibition of some isolates (Figure 3). Therefore, it is possible that isolates that survived for 48 h in BHI broth had high acid tolerance. There are several reports supporting the survival and adaptation of food pathogens such as *E. coli*, *Staphylococcus aureus*, *Listeria monocytogenes*, and *Salmonella* spp. to acidic environments [8, 14, 43–45]. In fact, outbreaks of diseases by *E. coli* O157 : H7 and *Salmonella enterica* in apple cider and orange juice (pH = 3.5 to 4.0) have been reported [9, 10]. This suggests that there is no guarantee for pathogens to be inhibited at stressful acid conditions. Therefore, the ability of lactic acid to induce the ATR in pathogens should be considered by food processors and other mechanisms put in place to prevent the growth of acid-resistant pathogens in acidified foods.

### 3.3. (GTG)_5_ Rep-PCR Genetic Fingerprinting and Identification

**Figure 4:**
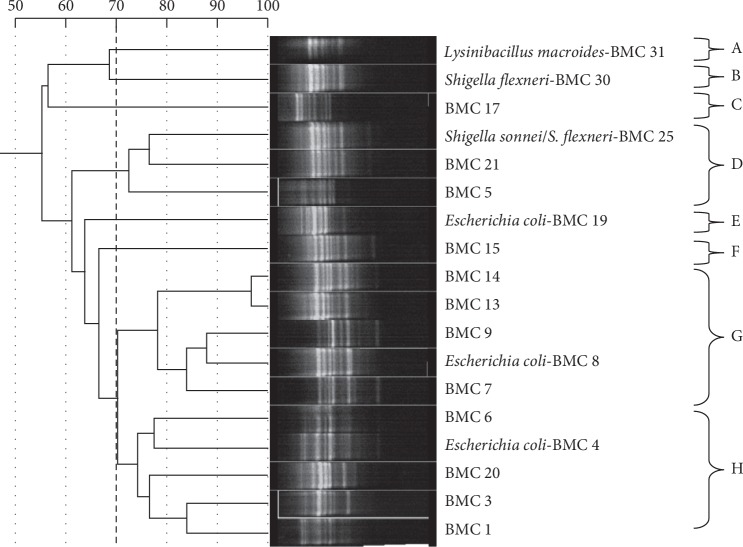
Cluster analysis of (GTG)_5_-Rep-PCR fingerprints of presumptive *E. coli* isolates. Dotted line shows clusters of isolates that showed 70% similarity which was the threshold for closely related isolates.

Based on their banding patterns, the different presumptive *E. coli* isolates were clustered based on 70% similarity as shown in Figure 4. Results from the 16S rRNA sequencing (Table 2) showed that three of the representative isolates from cluster analysis were closest relatives of *E. coli* (%ID = 97–98%, *E*value = 0.0). The rest were closest relatives of *Shigella sonnei*, *Shigella flexneri*, or *Lysinibacillus macroides* (%ID = 97–98%, *E* value = 0.0).

Repetitive extragenic palindromic (rep)-PCR has previously been shown to have high discriminatory power for *E. coli* strains of diverse origins [46, 47]. (GTG)_5_ Rep-PCR in particular is widely used as a high throughput genotyping tool for *E. coli* because the amplification of DNA sequences flanked between the polynucleotide (GTG)_5_ repetitive sequences generates typical DNA fingerprints for discriminating [48, 49]. Presumptive *E. coli* isolates were efficiently discriminated by (GTG)_5-_rep-PCR fingerprinting as shown in Figure 4. This means that *E. coli* isolates studied were from different sources and possess genetic variations and hence could express differences in virulence.

Some of the isolates with *E. coli* characteristic appearance on *E. coli*-Coliforms chromogenic agar were actually *Shigella* spp. (Table 2). In similarity with *E. coli*, some *Shigella* spp. have *β*-D glucuronidase which cleaves X-glucuronide in the chromogenic mixture of the agar resulting in the formation of blue colonies [50]. Like *Shigella*, *Lysinibacillus macroides* may also produce *β*-D glucuronidase enzyme, characteristic to *E. coli*. *Shigella* is known to cause *Shigellosis*, an acute invasive enteric infection clinically manifested by bloody diarrhea [51]. It is an endemic infection in many developing countries and is associated with considerable morbidity and mortality. For instance, between December 1999 and March 2000, about 4,000 cases of bloody diarrhea due to *Shigella dysenteria*e serotype 1 were reported in Kenama, Sierra Leone [52]. Although it is expected that lactic acid and other antimicrobial compounds in fermented foods can inhibit pathogens, *Shigella* has the potential of developing protective mechanisms towards acidic environments [53]. In contrary, *Lysinibacillus* spp. are pervasive bacteria rarely associated with human disease and merely regarded as environmental contaminants [54]. Nonetheless, some species such as *Lysinibacillus sphaericus* are reported to cause bacteremia in immune-compromised persons. For instance, the organism caused bacteremia in children with cancer and those undergoing bone marrow transplants in Italy [54]. Therefore, like *E. coli*, the presence of acid-resistant *Shigella* spp. and *Lysinibacillus* spp. in *Obushera* is a serious food safety concern and may indicate the survival of other potential pathogens in similar fermented cereal-based beverages.

### 3.4. Virulence Genes in *E. coli*

The *Stx* 2 gene was detected in *E. coli* BMC 4 and *E. coli* BMC 8, whereas none of the three genes considered in this study (*Stx 1*, *Stx 2*, and *eae*) were detected in *E. coli* BMC 19.

*E. coli* is among the most important causes of foodborne illness worldwide [55, 56]. Different virulent genes exist and are crucial for the pathogenicity of any bacterium. Among other serotypes of *E. coli*, the Shiga toxin producing *E. coli* (STEC) is the most important cause of foodborne diseases [57]. Shiga toxin producing *E. coli* (STEC) harbor many types of virulent factors particularly Shiga toxins (*Stx 1* and *Stx 2*), intimin (*eae*), and hemolysin (*hlyA*). These genes are responsible for settlement, adhesion, and invasion of the gastrointestinal mucosa by STEC [55, 56]. The toxins encoded by these genes inhibit protein synthesis and cause cell apoptosis [58]. They are also responsible for endothelial damage by causing cell swelling and separation from the basal membrane, fibrin, and thrombi. This narrows the capillary lumen and reduces blood flow to the glomeruli, resulting in renal failure [59]. In effect, STEC is responsible for diarrhea, hemolytic uremic syndrome, and hemorrhagic colitis [60]. The presence of virulence genes in our isolates agrees with other authors' results who reported similar genes in microorganisms isolated from acidic foods [23, 61, 62].

Byakika et al. [1] attributed the occurrence of *E. coli* and other undesirable microorganisms in *Obushera* to poor production hygiene. In addition to poor production hygiene, most of the *Obushera* processors do not pasteurize their product after fermentation. The raw materials, food processors, and packaging materials were also reported as possible sources of the contamination.

### 3.5. Antibiotic Susceptibility

Table 3 shows the number of *E. coli* that were susceptible, intermediately susceptible, and resistant to the different antibiotics. Two of the three isolates were resistant to ampicillin, amoxicillin, gentamicin, and trimethoprim-sulphamethoxazole. Only one of the isolates was resistant to ceftriaxone and tetracycline.

Table 4 shows the antibiotic resistant profiles and multiple antibiotic resistance (MAR) indices of the isolates, respectively. The MAR index for *E*. *coli* BMC 4, *E*. *coli* BMC 8, and *E*. *coli* BMC 19 was 0.00, 0.46, and 0.23, respectively.

Am: ampicillin 10 *μ*g; Ax: amoxicillin 25 *μ*g; Cro: ceftriaxone 30 *μ*g; Cn: gentamicin 10 *μ*g; Te: tetracycline 30 *μ*g; Stx: trimethoprim-sulphamethoxazole 25 *μ*g. A total of 13 antibiotics were tested.

O'Bryan et al. [63] reported that bacteria may possess innate resistance to antibiotics or may acquire it from other microorganisms. The acquisition of the resistance results from chromosomal mutation or gene transfer from one organism to another by plasmids. Bacterial pathogens can also employ biochemical types of resistance such as antibiotic inactivation, target modification, or removal of the antibiotic from the cell by efflux pumps. Antibiotic resistance is a serious global concern because a resistant infection can spread from one person to many others [64]. Antibiotic resistance genes can also be transferred between bacteria in the food chain. Walsh and Duffy [65] reported the transfer of ampicillin resistance genes from *Salmonella typhimurium* to *Salmonella agona* and *E. coli* K12 in pasteurized milk and minced beef.

The resistance of our isolates to the different antibiotics is in agreement with findings of other authors [23, 56, 66–71]. The occurrence of MAR among foodborne pathogens (Table 4) has also been previously documented [65, 69]. Illnesses associated with MAR microorganisms are challenging to treat [65]. The MAR index of *E. coli* BMC 8 was much greater than 0.2 possibly indicating that it originated from a high risk source of contamination where antibiotics are often used [72]. The resistance of *E. coli* BMC 8 to a number of human-based antibiotics (Table 2) implies that it was of anthropogenic origin. The link between human-based antibiotic resistance of foodborne pathogens and transmission by food handlers was previously suggested [23, 56, 67, 70]. This suggests that the processors may be involved in the cross-contamination of *Obushera* with antibiotic-resistant *E. coli*.

### 3.6. Antimicrobial Activity

**Figure 5:**
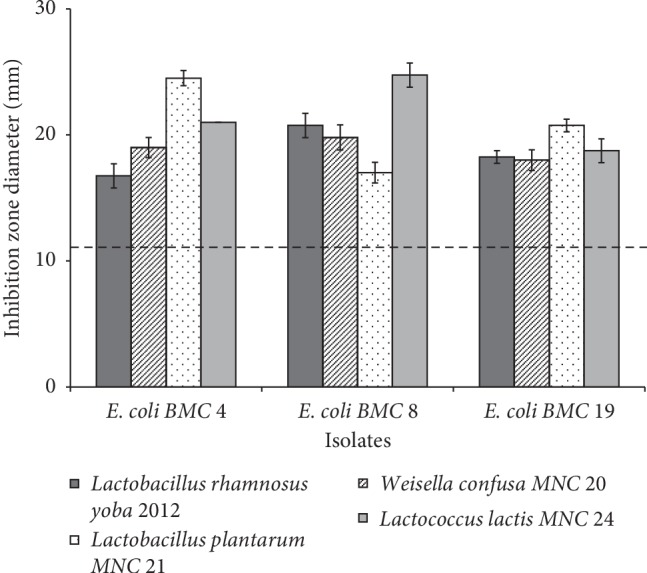
*E. coli* inhibition by lactic acid bacteria. Error bars show standard deviations of four independent determinations.

The extent of inhibition of *E. coli* by LAB is shown in Figure 5. All the *E. coli* were inhibited (inhibition zone diameter > 11 mm), but there was no specific trend observed. *Lb*. *plantarum* MNC 21 and *Lb. rhamnosus* yoba 2012 had the highest (24.5 mm) and lowest (16.8 mm) inhibition (*p* < 0.05) against *E. coli* BMC 4, respectively. In the same context, *L. lactis* MNC 24 exhibited the highest inhibition (24.8 mm) against *E. coli* BMC 8.

The use of LAB to inhibit food pathogens has been previously reported [73]. Lactic acid produced by LAB is the major organic compound in pathogen inhibition [4, 33]. *Lb. plantarum* MNC 21, *W. confusa* MNC 20, and *L. lactis* MNC 24 are fast and high lactic acid producers [2, 3]. Therefore, their ability to inhibit *E. coli* (Figure 5) is primarily attributed to the lactic acid. It is known that organic acids are most effective when in high levels, so for sufficient pathogen inhibition, large numbers of LAB are required [31, 74]. Previous studies have shown that pathogens do not survive well in prefermented foods in which LAB exist in large numbers (log 6-7) and pH ≤ 4 [21, 75]. In contrast, the same inhibitory effect may be jeopardized where LAB and pathogens are introduced in the food simultaneously [31, 76]. Therefore, the antimicrobial effect of acid fermentations should be seen as an adjunct to good hygiene practices rather than a substitute [31].

The objective of this study was to evaluate the antimicrobial effect of *Lb. plantarum* MNC 21, *L. lactis* MNC 24, *W. confusa* MNC 20, and *Lb. rhamnosus* yoba 2012 against acid-resistant, antibiotic-resistant, and potentially pathogenic *E*. *coli* isolated from *Obushera*. Findings indicated that the LAB starter cultures can inhibit growth of *E. coli* implying that they can be used to improve the safety of *Obushera* and other cereal-based beverages. The findings of this study should be validated by conducting food-based matrix studies. Further research should also explore the inhibitory effect of the LAB as co-cultures.

## Figures and Tables

**Table 1:** Primers and PCR conditions used for detection of virulence genes in *E. coli* isolated from *Obushera*.

Gene	Primers	PCR conditions	Base pairs	References
*stx 1*	5′CTTCGGTATCCTATTCCCGG3′ 3′GGATGCATCTCTGGTCATTG5′	30 cycles (94°C, 30 s; 56°C, 30 s; 72°C, 30 s)	448	[25]
*stx 2*	5′CCATGACAACGGACAGCAGTT3′ 3′CCTGTCAACTGAGCAGCACTTTG5′	30 cycles (94°C, 30 s; 56°C, 30 s; 72°C, 30 s)	779	[25]
*eae*	5′GTGGCGAATACTGGCGAGACT3′ 3′CCCCATTCTTTTTCACCGTCG5′	30 cycles (94°C, 30 s; 55°C, 30 s; 72°C, 1 min)	891	[26]

**Table 2:** Identities of presumptive *E. coli* isolates based on 16S rRNA sequencing.

Presumptive identity	Code	Closest relatives	Identity score, % sequence similarity^a^	*E* value
	BMC^*∗*^			
*Escherichia coli*	4, 8, 19	*Escherichia coli*	97-98	0.0
	25	*Shigella sonnei*, *Shigella flexneri*	97	0.0
	30	*Shigella flexneri*	97	0.0
	31	*Lysinibacillus macroides*	98	0.0

^a^Percent similarity with related sequences from the NCBI database. ^*∗*^Numerical code for a specific isolate.

**Table 3:** Prevalence of antibiotic susceptibility among *E. coli* (*n* = 3) isolated from *Obushera*.

Antibiotic	Susceptible	Intermediate	Resistant
Ampicillin (Amp) 10 *μ*g	1		2
Amoxicillin (Ax) 25 *μ*g	1		2
Amoxicillin-clavulanic acid (Amc) 30 *μ*g	3		
Cephalexin (Cl) 30 *μ*g	3		
Ceftriaxone (Cro) 30 *μ*g	2		1
Gentamicin (Cn) 10 *μ*g	1		2
Kanamycin (K) 30 *μ*g	1	2	
Tetracycline (Te) 30 *μ*g	2		1
Chloramphenicol (C) 30 *μ*g	3		
Ciprofloxacin (Cip) 5 *μ*g	3		
Levofloxacin (Lev) 15 *μ*g	3		
Trimethoprim-sulphamethoxazole (Stx) 25 *μ*g	1		2
Nitrofurantoin (F) 300 *μ*g	3		

**Table 4:** Antibiotic resistance profiles and multiple antibiotic resistance (MAR) indices of *E. coli* isolated from *Obushera*.

Isolate	Antibiotic resistant profile	Number of antibiotics	MAR index
*E. coli* BMC 4	—	—	0.00
*E. coli* BMC 8	AxAmCnCroStxTe	6	0.46
*E. coli* BMC 19	AmCnStx	3	0.23

## Data Availability

The data used to support the findings of this study are included within the article.
